# Combining perceptual regulation and exergaming for exercise prescription in low-active adults with and without cognitive impairment

**DOI:** 10.1186/s13102-018-0091-7

**Published:** 2018-01-30

**Authors:** Liam McAuliffe, Gaynor C. Parfitt, Roger G. Eston, Caitlin Gray, Hannah A. D. Keage, Ashleigh E. Smith

**Affiliations:** 1 Alliance for Research in Exercise Nutrition and Activity (ARENA), Sansom Institute for Health Research, School of Health Science, University of South Australia, GPO Box 2471, 108 North Terrace, Adelaide, SA 5001 Australia; 2 Cognitive Ageing and Impairment Neurosciences (CAIN) Laboratory, School of Psychology, Social Work and Social Policy, University of South Australia, St Bernards Rd Magill, Adelaide, SA 5072 Australia

**Keywords:** Ratings of perceived exertion, Mild cognitive impairment, Oxygen uptake, Affect, Ageing

## Abstract

**Background:**

Exercise adherence in already low-active older adults with and without mild cognitive impairment (MCI) remains low. Perceptual regulation and exergaming may facilitate future exercise behaviour by improving the affective experience, however evidence that this population can perceptually regulate is lacking. To explore this, we investigated 1) perceptual regulation of exercise intensity during either exergaming or regular ergometer cycling and 2) explored affective responses.

**Methods:**

Thirty-two low active older adults (73.9 ± 7.3 years, *n* = 16, 8 females) with or without MCI (70.9 ± 5.5 years, *n* = 16, 11 females) participated in a sub-maximal fitness assessment to determine ventilatory threshold (VT) and two experimental sessions (counterbalanced: exergaming or regular ergometer cycling). Experimental sessions consisted 21-min of continuous cycling with 7-min at each: RPE 9, 11 and 13. Oxygen consumption (VO_2_), heart rate (HR), and affect (Feeling Scale) were obtained throughout the exercise.

**Results:**

VO_2_ (*p* < 0.01) and HR (*p* < 0.01) increased linearly with RPE, but were not significantly different between exercise modes or cognitive groups. At RPE 13, participants worked above VT in both modes (exergaming: 115.7 ± 27.3; non-exergaming 114.1 ± 24.3 VO_2_ (%VT)). Regardless of cognitive group, affect declined significantly as RPE increased (*p* < 0.01). However on average, affect remained pleasant throughout and did not differ between exercise modes or cognitive groups.

**Conclusions:**

These results suggest low-active older adults can perceptually regulate exercise intensity, regardless of cognition or mode. At RPE 13, participants regulated above VT, at an intensity that improves cardiorespiratory fitness long-term, and affect remained positive in the majority of participants, which may support long-term physical activity adherence.

## Background

Australian physical activity (PA) guidelines indicate that as few as one in three older men and one in five older women (> 65 years) are considered sufficiently active (achieving at least 150 min moderate intensity activity per week) [1, 2]. In this population, regular PA may not only be preventative against cognitive decline and reduce the risk of future cognitive impairment [3–5], but might also slow the progression of an existing mild cognitive impairment (MCI) to dementia [6–8]. Despite this, one of the biggest challenges is engaging low-active older adults, both with and without cognitive impairment, in regular and sustainable PA. Evidence from one study demonstrates only 53% of older adults living with MCI adhere to a six month traditionally prescribed, moderate intensity exercise program; and as few as 25% maintain sufficient PA, six months after completion [9]. Taken together, these findings suggest traditional, moderate intensity exercise prescription approaches do not translate to long-term adherence, in this population. Therefore, new and novel exercise prescription approaches are needed to combat both physical inactivity and age-related cognitive decline.

One method of exercise prescription increasingly recognised as an effective way to promote long-term adherence is perceptual regulation of the exercise intensity [10]. Rather than an externally controlled intensity, such as percentage of age-predicted maximal heart rate (%HRmax), participants use effort perception (with the Borg 6–20 Rating of Perceived Exertion (RPE) Scale) and their internal framework to individually set the intensity [11]. At any point throughout the exercise, participants can increase or decrease their output to maintain the desired effort perception. When perceptually regulating at different RPEs, participants work at discretely different intensities with high repeatability and reliability [10, 12, 13]. Additionally, the process of perceptual regulation has been linked to increased exercise adherence [10]. It is likely, that the autonomy present when perceptually regulating, and the acute affective (pleasant/unpleasant) responses during exercise, underpin the increased adherence [14]. A growing body of evidence shows promise for the use of perceptual regulation in sedentary adults [15], clinical populations [16, 17] and sufficiently active older adults [18]. Preliminary evidence in active older adults with no cardiovascular disease risk factors other than age, supports the use of RPE and perceptual regulation during a fitness assessment to accurately predict cardiorespiratory fitness [18]. However, there is no current evidence for the use of RPE to regulate exercise intensity in low-active older adults, or those living with MCI, and indeed many clinicians and care workers indicate at least anecdotally, that low-active older adults and people with MCI do not understand how to regulate their exercise intensity using the RPE scale.

Another emerging exercise modality that may support long-term adherence is exergaming [19]. Exergaming combines physical exercise with a computer-simulated interactive game. Studies report greater improvements in cognitive outcomes (including executive function) occur following a three month exergaming intervention, compared to traditionally prescribed exercise in adults with MCI [20]. Interestingly participants who used exergaming also anecdotally reported increased enjoyment during exercise [20], which may support future adherence to the intervention. A systematic review of the literature further demonstrates increased physical and cognitive benefits of exergaming in older adults, compared to traditional exercise interventions [21]. Together these studies provide support for exergaming as an effective approach for exercise prescription in older low-active adults, with and without MCI.

Two models may partially explain the increased enjoyment with the use of exergaming: Ekkekakis’s [22] dual-mode model and Tenenbaum’s social cognitive theory of perceived and sustained effort [23]. Whilst not the same, both models propose a link between awareness of internal (physiological) sensations during exercise and affective responses, with a negative shift as the intensity increases. The dual-mode model [22], proposes affective responses are largely guided by cognitive processes and are uniformly positive at low intensities [22, 24]. However, as the intensity of exercise increases beyond a point of physiological steady state (defined as the ventilatory threshold (VT)), physiological cues dominate, and this is associated with a homogenous unpleasant response [25, 26]. Exergaming, at intensities around VT, may distract from unpleasant (physiological) sensations and lead to more pleasant affective responses and higher exercise outputs, particularly if perceptually regulating.

Therefore this study had two overarching aims. Firstly, we aimed to investigate if low-active older adults with and without MCI were able to perceptually-regulate their exercise intensity at three submaximal intensities (relative to RPE 9-very light, RPE 11-light and RPE 13-somewhat hard) during exergaming or non-exergaming. We hypothesised 1) that older adults, regardless of their cognitive ability, will be able to perceptually regulate exercise at the three intensities. and 2) exergaming mode will be associated with higher work rates compared to standard ergometer cycling. Similar to studies in other populations [10, 26], we also hypothesised 3) that work rate selected at RPE 13 will be around known physiological thresholds (VT);

Additionally, we aimed to investigate affective responses across the duration of the exercise sessions. We hypothesised 4) that affective responses will remain pleasant across each submaximal intensity but less at RPE 13 compared to RPE 9 and 11 and 5) the exergaming condition will be associated with more pleasant affective responses at each intensity compared to non-exergaming.

These findings will provide the first evidence for the combined use of perceptual regulation and exergaming in this at-risk population.

## Method

### Participants

Thirty-two insufficiently active older adults provided informed written consent and participated in the study (Table 1). Eligibility for participation was determined by self-reporting less than 150 min of moderate intensity PA per week [1]. Recruitment occurred through local newspaper advertisements targeting low-active older adults who were concerned about their memory. All experimental procedures were approved by the University of South Australia’s human research ethics committee and were performed in accordance with the ethical standards laid down in the Declaration of Helsinki.

**Table 1:** Participant characteristics

	MCI	Apparently healthy
Participants	16	16
Males	8	5
Females	8	11
Age (years)	74.13 ± 7.44	70.88 ± 5.29
Height (cm)	164.8 ± 9.6	164.2 ± 7.8
Weight (kg)	68.56 ± 9.32	70.30 ± 15.17
BMI(kg/m^2^)	25.26 ± 2.02	26.11 ± 4.17
Resting pulse (bpm)	69.6 ± 10.7	71.41 ± 9.0
Age predicted HR max (bpm)	155.1 ± 5.2	157.4 ± 3.7
Predicted VO_2 max_ (ml/min/kg)	26.5 ± 5.5	25.58 ± 6.2
Systolic blood pressure (mm/hg)	145.7 ± 20.2	134.0 ± 16.8
Diastolic blood pressure (mm/hg)	74.4 ± 9.0	70.1 ± 9.1
Body fat (%)	30.0 ± 7.8	34.2 ± 7.0
Fasting glucose (mmol/l)	4.04 ± 1.36	3.66 ± 0.76
High density lipids	1.43 ± 0.49	1.32 ± 0.38
Total cholesterol	4.39 ± 1.16	4.69 ± 0.98
VO_2_ at VT (ml/min/kg)	12.8 ± 2.3	12.2 ± 2.5
Heart rate at VT (bpm)	97.3 ± 9.3	97.1 ± 10.5
ACE-III score mean	81.7 ± 4.2*	92.5 ± 3.8*
ACE-III score median (range)	83 (75–87)	91 (88–99)

*P < 0.05 between cognitive groups

Scores from the Addenbrooke Cognitive Exam (ACE-III) were used to dichotomise participants into groups using a previously reported clinical cut-off [27–29] (apparently healthy (score ≥ 88/100) or MCI (score < 88)).

### Experimental protocol

Participants attended the laboratory on three separate occasions within a four-week period (once for a screening session and twice for the experimental sessions).

#### Screening session: Session 1

Initially, height, mass, percent body fat (Tanita BF-679 W bioelectric impedance analysis scale; Tanita Corporation, Tokyo, Japan), blood pressure and resting heart rate (Dinamap Pro 100 automated sphygmomanometer), fasted total and high density cholesterol, and blood glucose (CardioCheck PA Point-of-Care Device, Indianapolis, IN) were measured. Participants were then served a standardised breakfast of toast and cereal.

Following familiarisation with the Borg 6 to 20 Scale and the Feeling Scale (used to assess affective responses), participants completed an experimenter-controlled submaximal exercise test on a recumbent ergometer (Lode Corival Recumbent, Groningen, The Netherlands) to assess cardiorespiratory fitness and determine VT.

Oxygen uptake (VO_2_) was measured continuously via a breath-by-breath automatic gas exchange system (Cortex MetaAnalyzer 3B, Biophysik, and Cortex Metasoft 3.1 software, Leipzig, Germany). Heart rate was continuously monitored using a wireless chest strap telemetry system (Polar Electro T31, Kempele, Finland).

The exercise test began with a 2-min warm up and familiarisation. Participants were instructed to cycle between 60 and 70 rpm. The test began at 15 W or 25 W and increased in either 15 W or 25 W increments each minute. The minute-by-minute incremental increases in resistance (either 15 W or 25 W) were determined by the sex of the participants and self-perceived fitness levels, with the aim of achieving test completion between 5 and 12 min of exercise. Individual RPE and affect were obtained each minute, and the test was terminated when participants reported an RPE of 15.

#### Experimental sessions: Session 2 and 3:

The order of the experimental sessions were randomised and counterbalanced so half of the participants received the exergaming session first and half the non-exergaming.

Both the Feeling Scale and the Borg 6–20 RPE Scale were displayed in front of the participants. Participants were asked to report affect and RPE prior to exercise, every two minutes for the entire duration of the test and immediately following exercise completion. Heart rate and VO_2_ were recoded continuously throughout the duration of the exercise session.

Session two and three, commenced with a brief 2-min warm up on the Expresso HD Recumbent Bike. Participants were then instructed to cycle for 21 continuous minutes, consisting of seven minutes at RPE 9 (equivalent to a ‘very light’ intensity), seven minutes at RPE 11 (equivalent to a ‘light’ intensity) and seven minutes at RPE 13 (equivalent to a ‘some-what hard’ intensity) either using the ‘Track mode’ of the Expresso bike (exergaming) or with the screen blocked (non-exergaming).

The exergaming session required the participants to cycle and steer their “avatar” around a pre-defined track steering with the steering levers attached to the side of the seat, while maintaining the targeted exercise output. During the control session the screen was blocked out and participants were not required to steer, but the same cycle ergometer was used. All outputs were concealed from the participants during the testing.

Throughout each exercise session, participants were reminded they could change resistance, or cycling cadence at any point to maintain the required exercise intensity. A continuous 21-min exercise duration was chosen so that seven minutes could be spent at each intensity, which allowed for multiple measurements at each intensity and replicated a standard aerobic session.

#### Prediction of fitness and determination of ventilatory threshold

To predict cardiorespiratory fitness levels, VO_2_ was collated from the final 30 s of each minute from the submaximal exercise test and extrapolated with HR using linear regression to an age-predicted HR max (obtained from Tanaka [30] equation, 208–0.7*age). The VT was individually determined using a triangulation of the modified v-slope, ventilatory equivalents (VE) and excess CO_2_ methods previously described by Gaskill and colleagues [31]. For the v-slope method, VT was identified as the point at which there was a disproportionate increase in VCO_2_ compared to VO_2_. For the VE method, VT was identified as the exercise intensity corresponding to the first sustained disproportionate increase in VE/VO_2_ with no increase in VE/VCO_2_. For the excess CO_2_ method, VT was identified as the exercise intensity corresponding to an increase in excess CO_2_ from steady state. The three methods were graphed separately for each participant and VT identified by two independent raters (LM and AS). If there was a disagreement on the location of the VT, the data were independently assessed by a third rater (GP or CG). For all subsequent analyses, VO_2_ was expressed relative to VT and all HR data as a percentage of age-predicted HRmax [30].

### Statistical analysis

To investigate if older low-active adults could regulate exercise intensity using perceptual regulation (hypothesis 1 and 2) physiological variables (VO_2_ and HR) were analysed with separate three-way analysis of variances (ANOVAs). Within subject factors were mode (2 levels: exergaming or non-exergaming) and intensity (3 levels: RPE 9, 11 and 13). The between subjects factor was cognitive group (2 levels: Apparently healthy or MCI). To ascertain if work rate at RPE 13 was around VT (hypothesis 3) a one-way ANOVA was conducted to compare VO_2_ at VT from the submaximal graded exercise test to the average of VO_2_ during the final minute of RPE 13 (minute 20) of both the exergaming and non-exergaming modes. To explore the stabilisation of physiological responses (VO_2_ and HR) within each of the three submaximal intensities, the coefficient of variations were obtained for each intensity by averaging the VO_2_ (last 30 s) in two-minute blocks at each exercise intensity and then obtaining a ratio of standard deviation to the mean, expressed as a percentage. Coefficient of variations were also analysed with three-way ANOVA. Within subject factors were mode (2 levels: exergaming or non-exergaming) and intensity (3 levels: RPE 9, 11 and 13) and the between subjects factor was cognitive group (2 levels: Apparently healthy or MCI).

Affective responses (Feeling scale) were also analysed with a three-way ANOVA (hypothesis 4 and 5). Within subject factors were mode (2 levels: exergaming or non-exergaming) and intensity (3 levels: RPE 9, 11 and 13). The between subjects factor was cognitive group (2 levels: Apparently healthy or MCI).

Post Hoc tests with Bonferroni correction were conducted on all significant main effects or interactions. Normal distribution and homogeneity of variance of the data were assessed using the Kolmogorov-Smirnov test and Levene’s statistic, respectively. Effect sizes are presented as Eta squared (η^2^) [32]. In ANOVAs where assumptions of sphericity were violated, the critical value of F was adjusted using the Greenhouse-Geisser epsilon value. All data were analysed using the Statistical Package for Social Sciences (SPSS) version 22 software.

## Results

Thirty-one participants completed both non-exergaming and exergaming sessions, while one participant (apparently healthy group) was excluded for changes to their medication during the study. Three participants (1 apparently healthy, 2 MCI) did not have valid fitness data to predict VT or fitness, and this is reflected in the difference in the degrees of freedom for specific analyses. Sixteen participants scored equal to or below the clinical threshold of 88/100 on the ACE-III.

Table 1, illustrates the mean descriptive participant characteristics between the cognitive groups. There were no differences in age, predicted cardiorespiratory fitness or VT. However, as expected ACE-III score was lower in the MCI group (t _[30]_= − 7.984, *P* < 0.001).

### Physiological evidence consistent with the use of perceptual regulation

**Fig. 1:**
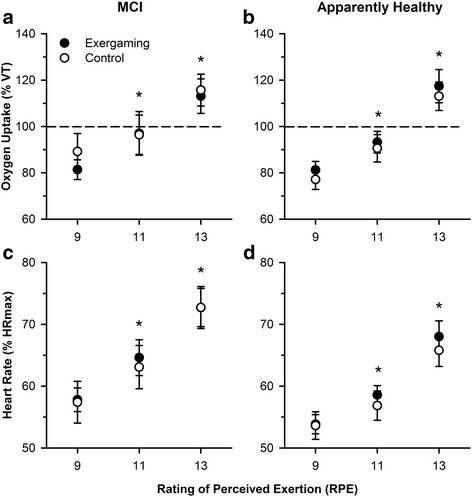
VO_2_ (**a** and **b**) and HR data (**c** and **d**) during 21 min of non-exergaming (open symbols) and exergaming (coloured symbols) in the MCI group (**a** and **c**) and the apparently healthy group (**b **and **d**). VO_2_ (% VT) and HR (% HR_max_) increased with each RPE level. There were no differences in either VO_2_ or HR between exercise modes (exergaming or non-exergaming). *P < 0.05, error bars indicate standard error of the mean (SEM)

In support of hypothesis 1, there was a step-wise increase in VO_2_ at each RPE (F _[2, 52]_ = 71.00, *P* < 0.001, η^2^ = 0.52; Fig. 1a and b) but no other main effects of mode (hypothesis 2), cognitive group or interactions with mode or cognitive group. Similarly, there was a step-wise increase in HR at each RPE (F _[1.3, 40.18]_ = 94.27, *P* < 0.01, *η*^2^ = 0.55, Fig. 1c and d) and no other main effects or interactions.

### Physiological evidence of working above ventilatory threshold at RPE 13

At RPE 13 (hypothesis 3), VO_2_ was significantly higher in the exergaming mode, compared to the VO_2_ at RPE 13 in the submaximal exercise test and the non-exergaming mode (F_[2,54]_ = 4.10, *P* = 0.02, *η*^2^ = 0.13). There was no significant difference between VO_2_ at VT in minute 20 of the non-exergaming and the VO_2_ at VT in the submaximal exercise test (*P* = 0.14).

The coefficient of variation analysis of physiological variables (VO_2_ and HR) revealed no main effects for intensity, mode, cognitive group or interactions (Table 2).

**Table 2:** Coefficient of variation (%) of physiological variables

		MCI		Apparently healthy	
		Exergaming	Non-Exergaming	Exergaming	Non-Exergaming
Oxygen uptake (VO_2_)					
RPE	9	7.8	10.8	7.9	9.2
	11	9.0	7.4	6.3	5.5
	13	7.4	7.8	6.3	6.7
Heart Rate (HR)					
RPE	9	3.3	3.7	3.4	3.6
	11	3.6	3.8	2.9	2.5
	13	3.8	4.2	4.8	3.6

### Affective responses during perceptual regulation

**Fig. 2:**
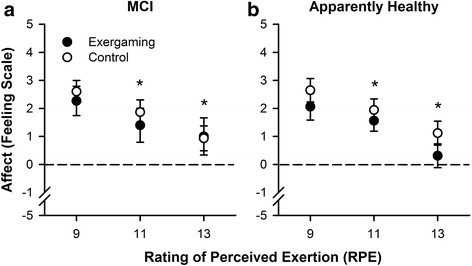
Affective responses across the 21-min of exergaming (coloured symbols) or non-exergaming (open symbols) in either MCI group (**a**) or apparently healthy (**b**). Affect significantly declined, but on average remained positive throughout the entire session. There was no difference in affective responses between exercise modes (exergaming or non-exergaming). *P < 0.05 across RPE levels, the error bars indicate standard error of the mean (SEM)

For affect (hypothesis 4 and 5) there was a main effect of intensity (F_[2, 58]_ = 38.15, *P* < 0.001, η^2^ = 0.28, Fig. 2), but no other main effects or interactions for mode or cognitive group. Affect declined from RPE 9 to RPE 11 (*P* = 0.002) and RPE 11 to RPE 13 (*P* < 0.001). Despite this, the mean group affective responses remained pleasant each: RPE 9, RPE 11 and RPE 13 (Table 3). During the final time point of each RPE level, a small proportion of participants reported below neutral responses (Table 3).

**Table 3:** Affective responses during perceptual regulation, mean ± SD (% participants reporting negative affect)

		MCI		Apparently healthy	
		Exergaming	Non-Exergaming	Exergaming	Non-Exergaming
RPE	9	2.25 ± 0.36 (12.5)	1.55±0.43 (12.5)	2.88 ± 0.43 (0)	2.80 ± 0.43 (0)
	11	1.41 ± 0.37 (0.25)	1.85 ± 0.23 (12.5)	1.78 ± 0.23 (0)	2.27 ± 0.27 (0)
	13	0.79 ± 0.57 (31.5)	1.08 ± 0.42 (25)	0.76 ± 0.48 (25)	1.35 ± 0.27 (12.4)

## Discussion

This study provides the first evidence that low-active older adults, regardless of MCI, are able to perceptually regulate their exercise intensity at three submaximal levels (RPE 9, 11 and 13). In line with other research [26], participants choose an intensity around their individual VT at RPE 11 and RPE 13, which can improve fitness long-term [10, 33]. Additionally, for the majority of participants, affective responses remained pleasant throughout. Unexpectedly, with the added stimulus of exergaming compared to non-exergaming we showed no differences in work rate or affect. Together these findings highlight the potential clinical benefit of using perceptual regulation in this at-risk population.

A future practical application of these findings for this population, might be an intervention using perceptual regulation anchored at RPE 11 or 13, to improve fitness. Indeed, in a similar population individualised training programs, externally prescribed at the VT were well tolerated, and led to greater improvements in fitness over a 12-week intervention, compared to traditional prescription at 50% HR reserve [17, 34]. A limitation of externally controlled individualised programs is the need to repeatedly assess/reassess the VT across the intervention, as participants improve fitness. Other studies, using perceptual regulation during interventions, albeit in younger populations, demonstrate participants regulate their output at higher work rates as they improve fitness (for the same RPE), thus negating the need to assess/reassess VT [10, 35].

A growing body of literature, demonstrates the importance of considering the affective response to exercise, alongside the intensity, to reduce exercise drop out [14, 36, 37]. In the present study, whilst affect consistently became less pleasant from RPE 9 to 11 and RPE 11 to 13, the majority of participants reported pleasant affective responses across the entire duration of the test. This characteristic shift in affect to become more unpleasant has been similarly reported in other populations when the intensity remains unchanged for a long duration [24, 38]. It is also important to note that towards the later stages of the sessions (RPE 13) 29% of participants reported an unpleasant affective response. One potential explanation for this response, is the long duration of the session (21 min) in a low-active population who do not exercise for extended periods, or additionally the higher work rate, which always occurred after 14 min of exercise at RPE 9 and 11. In light of these findings, we would be inclined to suggest it may be more appropriate at least initially, to perceptually regulate at RPE 11-light in this population. Anchoring exertion at a lower level would likely increase pleasant affective responses and reduce drop-out rates in an intervention, particularly in participants who report unpleasant affect at RPE 13. Future studies could also consider reordering the RPE levels, so participants finish the session at a lower RPE, which may uniformly be associated with a pleasant affect [39].

Unexpectedly, we saw no differences in work rate or affect in exergaming compared to non-exergaming. A potential explanation for this, is the immersion level of the particular game chosen provided insufficient distraction from internal physiological cues. Since there were few studies to base our design, a low level immersive option was pragmatically chosen to not over burden the participants cognitively. However, future studies using exergaming should consider immersion level. It is also possible that by asking participants to perceptually regulate, we may have inadvertently anchored the work rate, regardless of mode. Indeed, as reported in other studies, self-selection of exercise intensity [38], rather than perceptual regulation may be a more appropriate way to increase work rate and affect with exergaming.

### Study limitations

There are a few other study limitations that warrant discussion. Firstly, it is important to note this was an exploratory study with a small sample size and the sample was intentionally collected from older adults with memory concerns. This may affect the generalisation of results to the wider population of low active older adults. To be included in the study, participants were required to self-report as low-active, not meeting current activity of 150 min MVPA per week. However, actual physical activity was not verified and only a measurement of cardiovascular fitness was conducted. Both cognitive groups were well matched for fitness (Table 1), and predicted VO_2_max values fell below the fiftieth percentile for women and the thirtieth percentile for men when compared to the Australian fitness normative values indicating participants were likely insufficiently active [40]. However, it is possible that differences in the ability to perceptually regulate and affective responses to exercise in extremely physically inactive individuals may still exist. A larger sample size of extremely insufficiently active participants would be required to assess this.

## Conclusions

To combat growing physical inactivity, particularly in older adults with and without MCI, new and novel prescription approaches are needed [9]. Here, we provide the first evidence that low-active older adults (with and without MCI) can perceptually regulate their exercise intensity using the Borg 6–20 RPE scale and they do so at a similar intensity to other populations [12, 26, 41, 42]. Furthermore, the majority report a pleasant affective responses, which may support future adherence, long-term [14].

## Abbreviations

%HRmax: Percent heart rate maximum
ACE-III: Addenbrooke Cognitive Exam (Version 3)
ANOVA: Analysis of variance
CO_2_: Carbon dioxide
HR: Heart rate
MCI: Mild Cognitive Impairment
PA: Physical activity
RPE: Rating of perceived exertion
SPSS: Statistical Package for Social Sciences
VE: Ventilatory equivalent
VO_2_: Oxygen uptake
VT: Ventilatory threshold
W: Watts
